# Airway Management Practices in Burn Patients: A Protocol for a Scoping Review

**DOI:** 10.1111/aas.70327

**Published:** 2026-08-26

**Authors:** David Schiött Rasmussen, Tórálvur Mohr, Thomas Kronborg, Emma Atsuko Tsuchiya, Martin Risom Vestergaard, Mette Krag, Nicolai Haase, Christoffer Clemmesen, Rikke Holmgaard, Camilla Strøm

**Affiliations:** ^1^ Department of Anaesthesiology, Surgery and Trauma Centre Copenhagen University Hospital‐Rigshospitalet Copenhagen Denmark; ^2^ Department of Clinical Medicine University of Copenhagen Copenhagen Denmark; ^3^ Department of Intensive Care Copenhagen University Hospital—Rigshospitalet Copenhagen Denmark; ^4^ Department of Plastic Surgery and Burns Treatment, Centre of Head and Orthopedics Rigshospitalet Copenhagen Denmark

**Keywords:** airway extubation, burns, intubation, scoping review, tracheostomy

## Abstract

**Background:**

Loss of airway represents a serious clinical concern in the management of burn patients and frequently prompts early intubation. However, intubation itself carries inherent risks and is not invariably required for optimal care. Concern over the potentially severe consequences of airway compromise may lead clinicians to intubate more often than clinically warranted, thereby exposing patients to complications associated with airway management and mechanical ventilation. The lack of unified guidelines contributes to variation in practice, raising important questions about how burn victims are handled across different clinical settings. We aim to (1) provide a comprehensive overview of studies on airway management in burn patients, (2) describe available clinical decision tools or formal guidelines that support clinicians in deciding when burn‐injured patients should undergo endotracheal intubation, and (3) identify and synthesize reported outcomes with particular attention to adverse events associated with intubation in burn patients.

**Methods:**

We will conduct a scoping review in accordance with the Preferred Reporting Items for Systematic Reviews and Meta‐Analyses extension for Scoping Reviews (PRISMA‐ScR). We will systematically search Embase, PubMed, Scopus, Web of Science, and the Cochrane Library from 2011 onward, including randomized and non‐randomized trials, observational studies, and case series with ≥ 5 patients. We will use a Population, Concept, and Context‐based approach to define eligibility criteria: Population: burn patients (no age limitations); Concept: airway management of any kind and their associated outcomes; Context: timing of airway management, location, and healthcare provider of airway management. Two authors will independently screen studies, followed by full‐text assessment and data extraction. All reported clinical and procedural outcomes will be included. Findings will be synthesized descriptively to highlight key themes, evidence gaps, and areas of clinical uncertainty.

**Discussion:**

This review will provide an overview of existing evidence on airway management in burn patients, identify knowledge gaps, and help promote areas for future research.

## 1 Introduction

Airway management is a central concern in the care of patients with burn injuries. Decisions about tracheal intubation often need to be made rapidly, sometimes before the full extent of the burn injury is known. While early intubation may prevent airway obstruction from evolving edema, unnecessary intubation exposes patients to avoidable risks such as ventilator‐associated complications, prolonged intensive care unit stays, and delays to definitive burn care [1, 2, 3].

To help guide the decision to intubate, decision aids such as the American Burn Association (ABA) guidelines and the Denver criteria have been developed [4]. These tools provide a set of indications recommending intubation if identified during airway assessment [5]. However, these models may be overly sensitive, potentially leading to unnecessary intubations.

To the best of our knowledge, there is currently no comprehensive overview of adherence to these decision‐support models, nor of alternative airway management strategies or international consensus guidelines for airway management in burn patients. Evidence on this topic is scattered across prehospital, emergency department, intensive care, and operative settings, and varies widely in terminology, diagnostic criteria, and clinical thresholds for intervention. A scoping review is therefore well‐suited to map the breadth of available research, clarify how intubation decisions are made in burn populations, and highlight areas where evidence remains limited or inconsistent.

In this review, we aim to map the evidence on airway management in burn patients. Specifically, we aim to (1) provide a comprehensive overview of studies on airway management in burn patients, (2) describe available clinical decision tools or formal guidelines that support clinicians in deciding when burn‐injured patients should undergo endotracheal intubation, and (3) identify and synthesize reported outcomes with particular attention to adverse events associated with intubation in burn patients.

## 2 Methods

This protocol has been prepared in accordance with the Preferred Reporting Items for Systematic Reviews and Meta‐Analysis Protocols (PRISMA‐P) [6]. The scoping review will be conducted in accordance with the PRISMA extension for Scoping Reviews (PRISMA‐ScR) guidelines [7].

A Population, Concept, and Context (PCC) approach will define our eligibility criteria, in accordance with the Joanna Briggs Institute guidelines [8].

### 2.1 Study Type

We will include randomized trials, non‐randomized trials, and observational studies (cohort, case–control, and registry studies), as well as case series including ≥ 5 patients. Systematic reviews will be used for reference screening. Case series with fewer than five patients, case reports, conference abstracts, opinion pieces, and editorials will be excluded. Only peer‐reviewed articles will be considered. Articles not published in English or selected Scandinavian languages (Danish, Swedish, and Norwegian) will be translated using automated translation tools.

Due to the 2011 revision of the ABA guideline, introduced to standardize thresholds for intubation in burn patients [4], only studies published from 2011 onward will be included.

### 2.2 Population

We will include patients across all age groups with any type of burn injury (e.g., flame, flash, scald, explosion, chemical, electrical, frostbite, or contact). Both patients with and without inhalation injury will be included.

### 2.3 Concept

Any type of airway management (e.g., intubation, supraglottic devices, emergency front‐of neck access, tracheostomy, non‐invasive ventilation, or no intervention), as well as indications for airway intervention, including inhalation injury and decision aids such as the ABA guidelines [9] and Denver criteria [4] will be included.

### 2.4 Context

Airway management in any clinical setting will be included (e.g., prehospital, during transport, emergency department, intensive care unit, burn center, or other in‐hospital settings).

### 2.5 Search Strategy and Information Sources

The search strategy was developed in collaboration with an information specialist. We will search Embase, PubMed, Scopus, Web of Science, and the Cochrane Library.

The search strategy is available in Appendix S1.

### 2.6 Data Extraction

Following database searching, all identified records will be imported into the Covidence platform for screening and data management. Titles and abstracts will be screened independently by two authors against predefined eligibility criteria. Full‐text articles of potentially relevant studies will then be assessed by the main author. Disagreements will be resolved through discussion or, if necessary, consultation with a third author. Reasons for exclusion at the full‐text stage will be documented. The study selection process will be presented in a flow diagram in accordance with PRISMA‐ScR guideline [7].

Data will be extracted in accordance with the PRISMA‐ScR guideline [7]. If necessary, corresponding authors of included studies will be contacted to clarify reported data, missing information, or study methods. The main author will extract data from all included studies using a predefined data extraction form, which has been pilot‐tested on five studies meeting the eligibility criteria prior to formal data extraction. Data will be extracted on study characteristics, patient demographics, patient trajectory (e.g., prehospital care, burn unit admission, intensive care unit stay), use of intubation decision aids and other airway management decision‐support tools, use of airway‐related diagnostic or assessment tools, airway management and other airway interventions, and reported outcomes.

No formal risk‐of‐bias assessment will be conducted, but we will provide a narrative appraisal of methodological limitations across the included studies to contextualize the certainty and interpretability of the evidence.

### 2.7 Synthesis of Results

Results will be reported in a narrative, descriptive manner.

## 3 Discussion

This scoping review will provide a comprehensive overview of the existing evidence on airway management in burn patients and identify key gaps in the literature. Particular emphasis on intubation decision‐making, including available clinical guidelines and decision‐support tools, may offer insight into how clinicians currently approach airway management in this patient group. Furthermore, synthesizing reported outcomes—with a focus on intubation‐related adverse events—may help clarify the balance between timely intervention and potential overtreatment, and inform whether more robust decision‐support strategies are warranted. The strengths of the planned scoping review include the predefined protocol, the systematic search and compliance with PRISMA statements [7]. The planned review has some limitations. Consistent with scoping review methodology, we will not assess the risk of bias or certainty of evidence. Furthermore, we expect some clinical heterogeneity amongst the included studies that may limit comparability. As the search is time‐bound, emerging evidence published after completion of the search may not be captured.

## 4 Conclusion

This scoping review will provide a comprehensive overview of the existing evidence on airway management in burn patients and identify key gaps in the literature which will help guide future research priorities and support the development of more evidence‐informed approaches to airway management in burn care.

## Author Contributions

All authors have contributed to the conception and design of this protocol. David S. Rasmussen drafted the manuscript. Camilla Strøm is the garantor. All authors participated in the revision of the manuscript and approved the final product.

## Funding

The authors have nothing to report.

## Ethics Statement

The authors have nothing to report.

## Conflicts of Interest

The authors declare no conflicts of interest.

## Supporting information


**Appendix S1:** Exemplified search strategy.

## Data Availability

Data sharing not applicable to this article as no datasets were generated or analysed during the current study.
