# Supplementary material for: Airway Management Practices in Burn Patients: A Protocol for a Scoping Review
Source: Acta Anaesthesiol Scand. 2026 Aug 26;70(9):e70327. doi: 10.1111/aas.70327 (PMC13518085; doi:10.1111/aas.70327)
Supplement: Supplementary file 1 [file AAS-70-0-s001.docx]

# Appendix S1 – Exemplified Search Strategy

**Search to be performed in Medline search engine:**

| 1. | exp Burns/ |
| --- | --- |
| 2. | ((Scald* or Thermal* or Inhal* or Chemic* or Electric* or Frost* or Cryo* or Contact* or Surfac*) adj9 (burn* or Injur*)).mp. |
| 3. | 1 or 2 |
| 4. | exp Airway management/ |
| 5. | exp Ventilators, Mechanical/ |
| 6. | exp Bronchoscopy/ |
| 7. | 4 or 5 or 6 |
| 8. | (Intubat* or Extubat* or Tracheostom* or Tracheotom* or Ventila* or Bronchoscop*).mp. |
| 9. | ((Airway* or Burn*) adj3 (manag* or Interven* or protect* or handling)).mp. |
| 10. | 7 or 8 or 9 |
| 11. | exp Decision making/ |
| 12. | exp Guideline adherence/ |
| 13. | exp Guidelines as topic/ |
| 14. | exp Risk factors/ |
| 15. | exp Predictive value of tests/ |
| 16. | exp Severity of illness index/ |
| 17. | exp Clinical protocols/ |
| 18. | 11 or 12 or 13 or 14 or 15 or 16 or 17 |
| 19. | (Indicat* or Criteri* or Predict* or Factor* or Decision mak* or Decision-mak* or Guideline* or Protocol*).mp. |
| 20. | 18 or 19 |
| 21. | 3 and 10 and 20 |
| 22. | ("33707087" or "32856398" or "31982185" or "27777546" or "25423438" or "29123875").mp. |
| 23. | limit 21 to yr="2011 -Current" |
| 24. | 22 or 23 |
